# Risk Factors for Long-Term Mortality and Progressive Chronic Kidney Disease Associated With Acute Kidney Injury After Cardiac Surgery: Erratum

**DOI:** 10.1097/MD.0000000000007122

**Published:** 2017-06-02

In the article, “Risk Factors for Long-Term Mortality and Progressive Chronic Kidney Disease Associated With Acute Kidney Injury After Cardiac Surgery”,^[1]^ which appeared in Volume 94, Issue 45 of *Medicine*, Table 3 appeared incorrectly. The corrected table appears below.

**Table 3:**
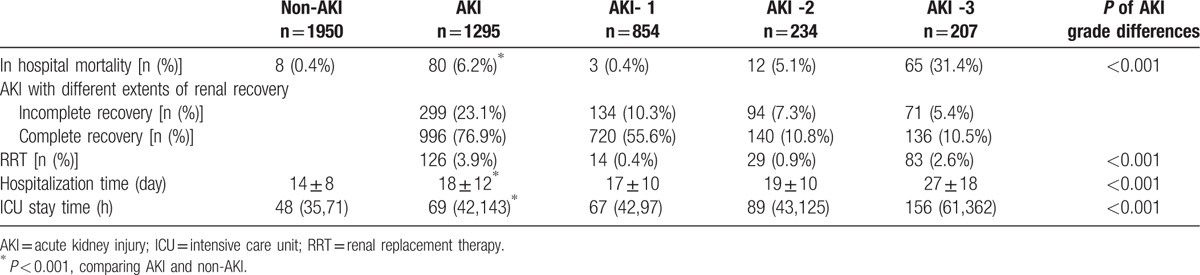
The short-term prognosis of AKI patients after cardiac surgery.
